# Amikacin use in critically ill patients requiring renal replacement therapy: the AMIDIAL-ICU study

**DOI:** 10.1186/s13613-025-01461-z

**Published:** 2025-03-26

**Authors:** Vincent Dupont, Bruno Mourvillier, Coralie Barbe, Vincent Legros, Mathieu Jozwiak, Hamid Merdji, Claire Dupuis, Hadrien Winiszewski, Antoine Marchalot, Guillaume Lacave, Mathilde Neuville, Anne Sagnier, François Barbier, Carine Thivilier, Stéphanie Ruiz, Roland Smonig, Jeremy Rosman, Laurent Argaud, Steven Grangé, Benjamine Sarton, Patrick Chillet, Guillaume Voiriot, Lukshe Kanagaratnam, Zoubir Djerada

**Affiliations:** 1 Centre Hospitalier Universitaire de Reims, 45 rue Cognacq Jay, Reims, 51092, +33326787641 France; 2 Université de Reims Champagne Ardenne, Reims, France; 3 Unité de Recherche Clinique Côte d’Azur, Centre Hospitalier Universitaire de Nice, Université Côte d’Azur, Nice, France; 4 Centre Hospitalier Universitaire de Strasbourg, Strasbourg, France; 5 Centre Hospitalier Universitaire de Clermont Ferrand, Clermont Ferrand, France; 6 Centre Hospitalier Universitaire de Besançon, Besançon, France; 7 Centre Hospitalier de Dieppe, Dieppe, France; 8 Centre Hospitalier de Versailles, Le Chesnay, France; 9 Hopital Foch, Suresnes, France; 10 Centre Hospitalier de Beauvais, Beauvais, France; 11 Centre Hospitalier Universitaire d’Orléans, Orléans, France; 12 Centre Hospitalier Universitaire de Nancy, Nancy, France; 13 Centre Hospitalier Universitaire de Toulouse, Toulouse, France; 14 Centre Hospitalier de Lorient, Lorient, France; 15 Centre Hospitalier Intercommunal Nord Ardennes, Charleville-Mézières, France; 16 Centre Hospitalier Universitaire de Lyon, Lyon, France; 17 Centre Hospitalier Universitaire de Rouen, Rouen, France; 18 Centre Hospitalier de Chalons en Champagne, Chalons en Champagne, France; 19 Service de Médecine Intensive Réanimation, Sorbonne Université, Centre de Recherche Saint-Antoine UMRS_938 INSERM, Assistance Publique– Hôpitaux de Paris, Hôpital Tenon, Paris, France

**Keywords:** Amikacin, Aminoglycoside, Renal replacement therapy, Intensive care unit, Pharmacokinetic

## Abstract

**Background:**

Acute kidney injury (AKI) requiring renal replacement therapy (RRT) is common in intensive care units (ICUs), yet optimal amikacin dosing in this context remains poorly understood.

**Methods:**

We conducted a prospective observational study across 18 French hospitals from April 2020 to January 2022. Adult ICU patients (aged > 18 years) receiving their first amikacin dose while on RRT were included. Data on demographics, RRT modalities, amikacin dosing, and therapeutic drug monitoring were collected. Using a pharmacokinetic modeling approach, we evaluated various amikacin regimens and simulated target attainment probabilities across different minimum inhibitory concentrations (MICs).

**Results:**

A total of 111 patients were included, with approximately two-thirds receiving continuous RRT. The median amikacin dose was 27 (25–30) mg/kg. Amikacin peak (Cmax) and trough concentrations were monitored in 53 (47.8%) and 76 (68.5%) patients, respectively. Continuous RRT and a history of chronic kidney disease reduced dialytic clearance. For a MIC ≤ 4 mg/L, a 15 mg/kg amikacin dose achieved Cmax/MIC and AUC/MIC targets in ≥ 90% of patients on intermittent dialysis, while 20 mg/kg was required for those on continuous dialysis. For a MIC = 8 mg/L, a 30 mg/kg dose was necessary to achieve Cmax/MIC ≥ 8.

**Conclusions:**

Our findings highlight suboptimal adherence to amikacin monitoring guidelines in ICU patients on RRT. Using pharmacokinetic modeling, we identified amikacin dosing recommendations ranging from 15 to 35 mg/kg to optimize efficacy and minimize risks, depending on MIC and dialysis modality.

**Supplementary Information:**

The online version contains supplementary material available at 10.1186/s13613-025-01461-z.

## Introduction

Aminoglycosides are broad-spectrum bactericidal antibiotics widely used in intensive care unit (ICU) settings for managing severe infections [1]. Among them, amikacin is considered the preferred choice due to its potent bactericidal activity against most aerobic gram-negative bacilli, including *Acinetobacter baumannii* and *Pseudomonas aeruginosa* [2]. However, amikacin’s narrow therapeutic index raises significant challenges, particularly in critically ill patients with altered pharmacokinetics [3, 4].

Current guidelines recommend: early administration of a single intravenous amikacin dose of 20–30 mg/kg over 30 min, monitoring of amikacin peak concentration (C_max_) after the first dose in severely ill patients with potential pharmacokinetic alterations, and monitoring of trough concentration (C_min_) in cases of renal impairment to mitigate the risk of toxicity [1, 5]. Pharmacokinetic targets include a C_max_/Minimum Inhibitory Concentration (MIC) > 8, an Area Under the Curve (AUC) within the first 24 h/MIC > 75, and a C_min_ <2.5 mg/L.

Acute kidney injury (AKI) is a frequent complication in ICU patients, requiring renal replacement therapy (RRT) in approximately one-third of cases. However, amikacin management guidelines and data on optimal dosing regimens in critically ill patients undergoing RRT remain limited [6, 7].

This study aimed to evaluate amikacin prescription and monitoring practices in ICU patients receiving RRT and to identify optimized amikacin dosing regimens using population pharmacokinetic modeling and Monte Carlo simulations.

## Methods

### Setting

We conducted a prospective multicenter observational study across 18 hospitals in France between April 2020 and January 2022, including 11 university hospitals (Reims, Clermont-Ferrand, Orléans, Rouen, Besançon, Lyon, Nancy, Nice, Strasbourg, Toulouse, Paris) and 7 non-teaching hospitals (Beauvais, Dieppe, Châlons-en-Champagne, Lorient, Versailles, Suresnes, Charleville-Mézières). Data were collected prospectively by local investigators using a standardized electronic form recorded in an online database (CleanWeb^®^). Informed consent was obtained from all participants or their representatives. The study was approved by the ethics committee (CPP Sud-Ouest et Outremer 2, 2020) and registered on ClinicalTrials.gov (NCT04322019, March 26, 2020).

### Study population

Inclusion criteria were: (i) adult patients aged over18 years (ii), admitted in a participating ICU (iii), initiation of amikacin treatment (with no prior amikacin injection in the preceding 7 days), and (iv) ongoing RRT at the time of amikacin injection or RRT initiation within 24 h of the first administration to account for a broader range of conditions and practices.

Due to the descriptive purpose of our primary objective, no formal sample size calculation was performed. Instead, we included all consecutive eligible patients from each participating center during the predefined inclusion period. However, to homogenize the contributions from each site and minimize overrepresentation by larger centers, we deliberately limited the number of patients per center to 10.

### Data collection

Demographic and clinical data were collected at enrollment, including age, sex, weight, and comorbidities such as chronic heart failure, chronic kidney disease (CKD; defined as baseline eGFR < 60 mL/min/1.73 m² using CKD-EPI), cancer, hemopathy, HIV infection, chronic obstructive pulmonary disease, or diabetes. Illness severity scores (SAPS II, SOFA), use of vasopressors, mechanical ventilation, or extracorporeal life support, 24-hour fluid balance, and plasma creatinine and albumin levels were recorded at the time of amikacin injection. Amikacin dosing and monitoring data, including timing and values of amikacin C_max_ and C_min_, were documented when available. RRT modality, timing relative to amikacin administration, infection characteristics (source, bacteremia, pathogen identification), and concurrent antimicrobial therapies were also recorded. Outcomes such as creatinine levels, RRT dependence, and mortality were assessed at Day *28.*

### Population pharmacokinetic modeling

A population pharmacokinetic model was developed to characterize the relationship between amikacin dosing and plasma concentrations. The modeling was performed using Monolix version 2023R1 (Lixoft, Antony, France). Parameter estimation was carried out using the stochastic approximation expectation-maximization algorithm, in conjunction with a Markov chain Monte Carlo procedure, to compute the maximum likelihood values [8, 9].

### Population pharmacokinetic covariate screening

We compared one-, two-, and three-compartment models with zero-order absorption and first-order elimination. To account for dialysis, total clearance (Cl) was modeled as physiological residual clearance when dialysis was off and as the sum of residual clearance and dialytic clearance (Cld) when dialysis was ongoing, using an “if/else” approach. This approach allows to consider both the dialysis event and its real-time duration. To capture variability between patients, we used an exponential model: *θ*_i_ = *θ*_TV_ × exp(*η*_i_), where *θ*_i_ represents the individual parameter, *θ*_TV_​ the typical parameter value, and *η*_i_​ the random effect for each patient. Random effects were assumed to follow a normal distribution with a mean of 0 and variance parameterized as a diagonal matrix. Correlations between random effects were also tested. Fixed parameters were modeled with a lognormal distribution.

Residual variability was assessed using different error models (constant, proportional, or combined). The model was evaluated using the Corrected Bayesian Information Criterion (BIC). Log-likelihood estimation used 20,000 Monte Carlo importance sampling iterations.

After selecting the best structural model, we evaluated demographic and clinical covariates (age, weight, dialysis status…), as described in the data collection section, to explain variability in amikacin pharmacokinetics. Covariates were included if they significantly reduced variability, improved model fit (lower BIC), and had a 95% confidence interval (CI) that excluded zero (*p* < 0.05). Prediction-corrected visual predictive checks were conducted with 10,000 simulations [9]. The 95% CI for all model parameters were estimated using a bootstrapping method with 1,000 resamples [10].

### Probability of target attainment

Using the final pharmacokinetic model, we simulated 10,000 amikacin clearance profiles for dosing regimens of 15, 20, 25, 30, and 35 mg/kg. Recognizing that total body weight (TBW)-based dosing can lead to unexpected high plasma concentrations, especially in obese patients [11], we developed two models incorporating either TBW or adjusted body weight (ABW) as a covariate for the volume of distribution. ABW was calculated using the formula: ABW = IBW + 0.38×(TBW − IBW), where IBW is the ideal body weight [12].

Monte Carlo simulations (*n* = 10,000), performed with Simulx version 2023R1 (Lixoft, Antony, France), evaluated the probability of target attainment (PTA) for key pharmacodynamic targets: C_max_/MIC ≥ 8 and AUC/MIC ≥ 75 within the first 24 h of treatment. Simulations included various MIC ranging from 1 to 16 mg/L, using a TBW distribution with a median of 84.1 kg in patients with or without history of CKD treated with continuous (24 h per day) or intermittent (4 h per day) dialysis [7].

### Statistical analysis

Statistical analyses were conducted using R software (v4.1.1, The R Foundation for Statistical Computing). Normality of continuous variables was assessed using the Shapiro–Wilk test. Gaussian-distributed variables are presented as mean ± standard deviation, while non-Gaussian variables are reported as median (interquartile range). Categorical variables are expressed as counts (percentages). Graphs were generated using Monolix (version 2023R1, Lixoft), Simulx (version 2023R1, Lixoft), or R (v4.1.1).

## Results

### Patients

A total of 111 critically ill patients were included in the study (Supplemental Figs. 1 and 2). Baseline characteristics are summarized in Table 1. Patients with identified source of infection presented with intra-abdominal (*n* = 9), respiratory (*n* = 8), urinary tract (*n* = 6), catheter-associated (*n* = 6), or skin and soft tissue infections (*n* = 2), with bacteremia in 39 (35.1%) cases. Pathogens were identified in 75 (67.6%) patients, predominantly Gram-negative bacilli (Supplemental Table 1).

**Table 1:** Characteristics of included patients

Variables	All patients (*n* = 111)
Age, years	66(56–73)
Male, *n*(%)	77(69.4)
BMI, kg/m^2^	30(25–33)
Admission type, *n*(%)	
Medical	86(77.5)
Scheduled surgery	6(5.4)
Urgent surgery	19 (17.1)
SAPS II	61(49–73)
SOFA score	12(10–15)
Comorbidities, *n*(%)	
CKD	43(38.7)
ESRD	12(10.8)
Heart failure	18(16.2)
Diabetes	45(40.5)
COPD	14(12.6)
Active solid cancer	13 (11.7)
Blood cancer	9 (8.1)
HIV	1 (0.9)
Characteristics at the time of injection	
Serum Albumin, g/L	21(18–26)
24 h Fluid balance, mL	1158(68-2448)
Vasopressor, *n*(%)	99(89.2)
Mechanical ventilation, *n*(%)	91(81.2)
ECLS, *n*(%)	11(9.9)
RRT at the time of injection, *n*(%)	41(36.9)
RRT within 24 h after injection, *n*(%)	70(63.1)
Time from injection to RRT, h	7(3–17)
Day 28 outcome, *n*(%)	
Plasma creatinine, umol/L	107(80–176)
RRT dependance	37(33.3)
Death	54(48.6)

Variables are presented as median(interquartile range) or number(percentage) as appropriate. BMI, body mass index; SAPS, simplified acute physiology score; SOFA, sequential organ failure assessment; CKD, chronic kidney disease; ESRD, end stage renal disease; COPD, chronic obstructive pulmonary disease; HIV, human immunodeficiency virus; ECLS, extracorporeal life support; eGFR, estimated glomerular filtration rate; RRT, renal replacement therapy

Two-thirds of patients received continuous RRT, initiated within 24 h after amikacin injection in 70 (63.1%) patients, with a median time hours between injection and RRT initiation of 0 (0–7.5) for continuous and 5.4 (1.98–15.99) for intermittent technique. Median dialysis duration was 49 (24–67) hours for continuous RRT and 4.5 [4, 5, 6, 7] hours for intermittent RRT (Supplemental Table 2).

### Amikacin management

The median amikacin loading dose was 27 (25–30) mg/kg (Table 2). C_max_ was measured in 53 (47.8%) patients, achieving levels > 60 mg/L in 47 (88.7%) cases. C_min_ was measured in 76 (68.5%) patients at a median time of 24 (23–28) hours post-injection, with only 4 (5.4%) patients achieving levels < 2.5 mg/L within 24 h. Fifteen (13.5%) patients received more than one dose; in these cases, amikacin was re-administered after a C_min_ <2.5 mg/L in one patient, after a C_min_ >2.5 mg/L in 10 patients, and without prior C_min_ measurement in 4 patients.

**Table 2:** Characteristics of Amikacin management

Variables	All patients (*n* = 111)
Amikacin dose, g	2.25 (2-2.5)
Amikacin dose, mg/kg	27(25–30)
C_max_ measurement, *n*(%)	53(47.8)
Time from injection to C_max_ measurement, min	60(60–75)
C_max_, mg/L	85(68–108)
C_max_ >60 mg/L, *n*(%)	47(88.7)
C_min_ measurement, *n*(%)	76(68.5)
Time from injection to C_min_ measurement, h	24(23–28)
C_min_, mg/L	14(9–24)
C_min_ <2.5 mg/L, *n*(%)	4(5.4)

Variables are presented as median(interquartile range) or number(percentage) as appropriate. C_max_, peak concentration; C_min_, trough concentration. All amikacin doses were administered as 30-minute intravenous injections

### Pharmacokinetic model Building

We analyzed 190 plasma values, including 85 patients with complete RRT timing data. A two-compartment model with zero-order absorption and first-order elimination was selected as the final structural model with a BIC of 1357.95. Covariates influencing Cld included continuous RRT modality and CKD history. Inclusion of RRT parameters as a covariate for Cld reduced between-subject variability from 62.68 to 56.05% (*p* = 0.006). Similarly, incorporating CKD history as a covariate further decreased between-subject variability from 56.05 to 38.67% (*p* = 0.01). The median amikacin Cld during continuous dialysis was 0.35 L/h in patients with preexisting CKD and 0.69 L/h in those without CKD. For intermittent dialysis, the median amikacin Cld was 0.85 L/h in patients with preexisting CKD and 1.68 L/h in those without CKD.

$$\begin{gathered}\:{\text{log}}\left( {Cl{d_i}} \right) = {\text{log}}\left( {Cl{d_{pop}}} \right) - 0.89 \hfill \\\,\,\,\,\,\,\,\,\,\,\,\,\,\,\,\,\,\,\,\,\,\,\, \times \:\left[ {{\text{Continous}}\:{\text{RRT}}\:{\text{modality}} = {\text{Yes}}} \right]\: - 0.68 \times \:[{\text{CKD}} = {\text{Yes}}] + {\eta _{Cld}} \hfill \\ \end{gathered} $$

To address variations in the volume of distribution within the study population, we developed two distinct final models (Supplemental Tables 3 and 4): one incorporating TBW and the other ABW. Both TBW and ABW demonstrated a positive correlation with the volume of distribution.

$$\:{\text{log}}\left( {V{1_i}} \right) = {\text{log}}\left( {V{1_{pop}}} \right) + 0.71 \times \:log\left( {\frac{{{\text{TBW}}}}{{84.97}}} \right) + {\eta _{V1d}}$$

$$\:{\text{log}}\left( {V{1_i}} \right) = {\text{log}}\left( {V{1_{pop}}} \right) + 0.95 \times \:log\left( {\frac{{{\text{ABW}}}}{{68.31}}} \right) + {\eta _{V1d}}$$

The inclusion of these covariates reduced between-subject variability to 33.18% for the TBW-based model (*p* = 0.002) and 33% for the ABW-based model (*p* < 0.001), respectively.

#### Model validation

Both models demonstrated strong correlations between observed and predicted amikacin concentrations (Spearman *r* = 0.989, *p* < 0.001, Supplemental Fig. 3). Residuals and metrics were symmetrical, normally distributed, and not significantly different from zero (*p* = 0.15–0.28) (Supplemental Fig. 4). Over 99% of the observed values fell within the prediction intervals, confirming the model’s accuracy in describing the observed data (Supplemental Fig. 5). Predictive checks and bootstrap analysis confirmed robustness, with 100% convergence and consistent parameter estimates.

#### Dosing simulations

**Fig. 1:**
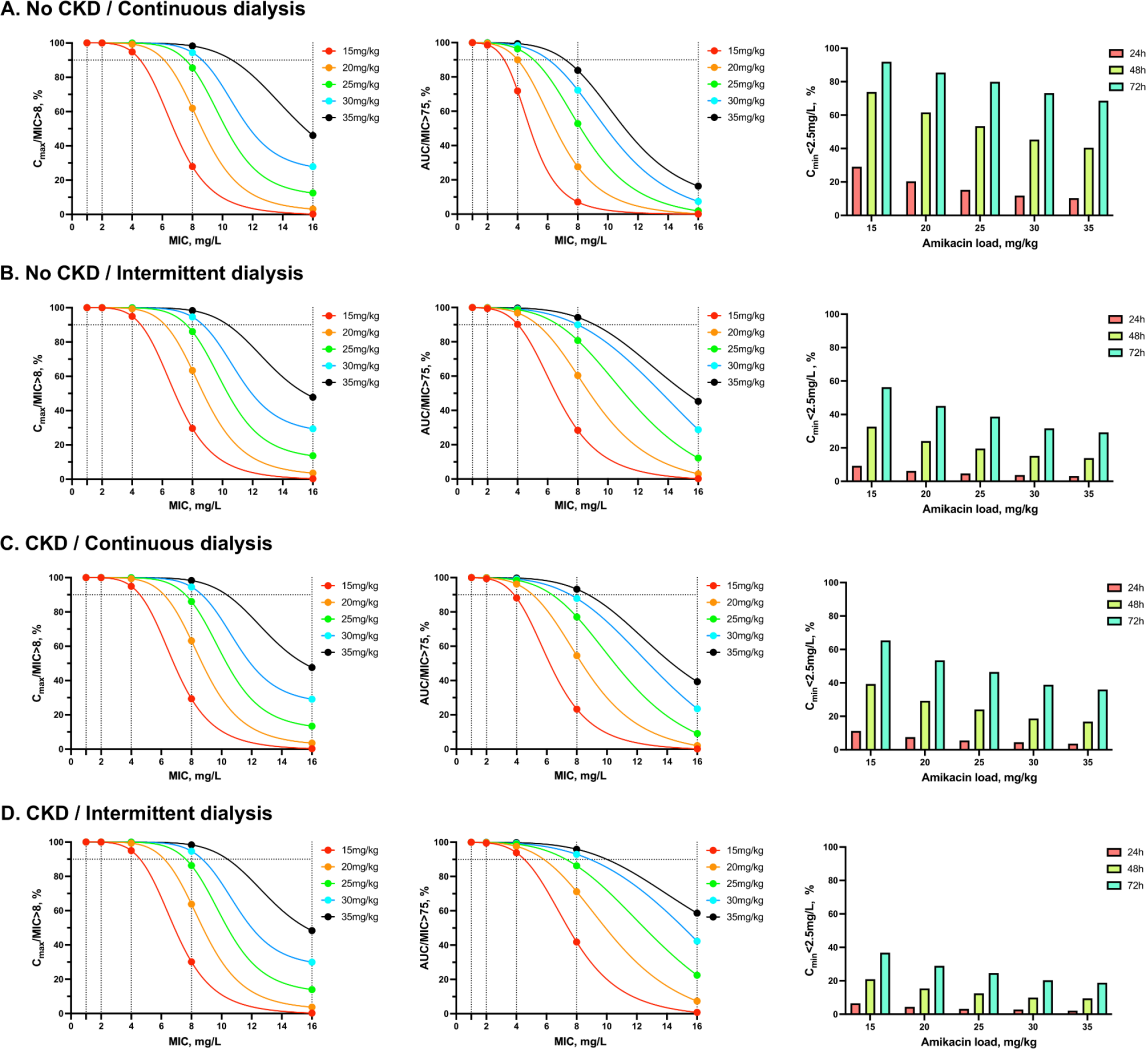
Probabilities of amikacin pharmacokinetic targets achievement in critically ill patients undergoing renal replacement therapy using total body weight. The probabilities to achieve a maximum concentration (C_max_) / minimum inhibitory concentration (MIC) ratio > 8, an area under the curve (AUC) within the first 24 h / MIC > 75 and a minimum concentration < 2.5 mg/L at 24-48-72 h in patients with a history of chronic kidney disease (CKD) treated with continuous (**A**) or intermittent (**B**) dialysis and patients without history of CKD treated with continuous (**C**) or intermittent (**D**) dialysis are depicted, based on Monte Carlo simulation

Simulations assessed target attainment probabilities for C_max_/MIC > 8, AUC/MIC > 75, and C_min_ <2.5 mg/L at various amikacin doses. For instance, for a MIC ≤ 4 mg/L:

- Using TBW-based dosing, a 15 mg/kg amikacin load achieved targets in ≥ 90% of intermittent RRT patients, while a 20 mg/kg dosage was required for continuous RRT. For an 8 mg/L MIC, 30 mg/kg was necessary (Fig. 1, Supplemental Table 5);
- Using ABW-based dosing, a 20 mg/kg amikacin load was adequate for intermittent RRT, while a 25–30 mg/kg dosage was needed for continuous RRT depending on CKD status. For an 8 mg/L MIC, 35 mg/kg was required (Supplemental Fig. 6, Supplemental Table 6).

Regardless of CKD status, RRT modality, or amikacin level, the probability of achieving a C_min_ < 2.5 mg/L at 24, 48, and 72 h was relatively low and showed an inverse correlation with amikacin dosage. This probability was higher in patients without a history of CKD compared to those with CKD and in patients undergoing continuous dialysis compared to those treated with intermittent dialysis (Fig. 1).

## Discussion

In this prospective multicentric cohort of ICU patients requiring RRT, the median amikacin dose adhered to current recommendations (20–30 mg/kg) despite underlying kidney impairment [1]. However, C_max_ was measured in fewer than half of the patients, and C_min_ was recorded in only about two-thirds. These observations suggest poor compliance with current amikacin monitoring guidelines in ICU patients undergoing RRT. Furthermore, when C_min_ was measured, it rarely met target levels, indicating potential prolonged exposure to high amikacin concentrations. Since amikacin overexposure is well-established as a cause of nephrotoxicity [13], identifying optimal dosing regimens to achieve pharmacokinetic targets while avoiding iatrogenic toxicity is crucial for managing this high-risk population, particularly those already experiencing acute kidney injury.

Using our database and pharmacokinetic modeling approach, we simulated various amikacin dosing regimens based on either TBW or ABW. Across all dosing strategies, the likelihood of achieving a C_min_ < 2.5 mg/L at 24, 48, or 72 h was consistently low and inversely correlated with amikacin dosage. As previously reported, the C_max_/MIC ≥ 8 target is most strongly associated with clinical outcomes in critically ill patients with Gram-negative bacilli infections [14]. The amikacin doses required to achieve C_max_/MIC ≥ 8 and AUC/MIC ≥ 75 in > 90% of cases ranged from 15 mg/kg to 35 mg/kg, depending on the MIC, CKD history, and RRT modality. These findings highlight the need for personalized amikacin dosing strategies in ICU patients requiring RRT to balance efficacy and safety.

The predominant pathogens in our cohort were *Escherichia coli*, *Klebsiella pneumoniae*, and *Pseudomonas aeruginosa*. According to the European Committee on Antimicrobial Susceptibility Testing data, the prevalence of an MIC ≥ 8 mg/L for these pathogens is 4.6%, 9.0%, and 22.5%, respectively. Therefore, higher amikacin dose should be considered, particularly when *Pseudomonas aeruginosa* is suspected or confirmed.

To our knowledge, no prior studies have directly compared the effects of intermittent versus continuous RRT modalities on amikacin pharmacokinetics in ICU patients. Data from animal models suggest that a 4-hour intermittent dialysis session initiated 2 h after amikacin administration could significantly enhance amikacin clearance (2.14 L/h), thereby potentially reducing renal toxicity [15]. However, reported amikacin clearance rates during continuous RRT in ICU patients vary widely (0.53 to 5.34 L/h) across studies. Beyond the intermittent vs. continuous debate, dialysis parameters, such as modality and filter type, likely influence amikacin clearance [16, 17, 18, 19, 20, 21]. Earlier studies suggested that diffusive methods could enhance amikacin clearance (+ 1.42 L/h), while more recent comparisons of continuous hemofiltration and continuous hemodiafiltration revealed a non-significant trend toward higher amikacin elimination in the latter group, despite similar effluent volumes [7, 20].

Interestingly, despite higher dialytic clearance rates with intermittent dialysis, our findings indicated delayed amikacin elimination with this modality. This paradox may be attributed to the shorter session durations of intermittent dialysis. Our results suggest that the prolonged duration of continuous dialysis compensates for its lower instantaneous clearance compared to intermittent techniques and extended intermittent dialysis sessions may provide the best risk-benefit balance. Additionally, continuous dialysis often employs polyacrylonitrile membranes, which are known to adsorb amikacin more effectively than polyamide membranes [22]. Future studies should explore the impact of dialysis membrane composition on amikacin pharmacokinetics in ICU patients. The only other factor independently associated with reduced amikacin clearance was a history of CKD, a result consistent with previous reports [23].

This study has several limitations. First, while the prospective multicentric design ensured broad representativity of the target population, variability in real-world data collection may have influenced pharmacokinetic modeling. This reflects the study’s dual objectives, characterizing real-life amikacin management and identifying optimized regimens, but it also introduced inconsistencies in patient monitoring. Notably, we did not collect data on the specific dialysis membranes used, which could significantly influence amikacin clearance. Additionally, amikacin MIC values for pathogens identified in our cohort were unavailable, limiting our ability to calculate real-world C_max_/MIC ratios. Finally, while the C_max_/MIC ratio is widely accepted as an indicator of maximal bactericidal activity against Gram-negative pathogens, it does not necessarily account for adequate tissue-level antimicrobial concentrations.

## Conclusions

Our findings indicate that day-to-day clinical practice demonstrates poor compliance with amikacin monitoring guidelines in ICU patients requiring RRT. Despite recommendations to monitor amikacin peak and trough concentrations to ensure efficacy and minimize toxicity, such practices remain inconsistently implemented. Using a pharmacokinetic modeling approach, we identified a range of amikacin doses (15–35 mg/kg) to optimize the risk-benefit balance. These doses were influenced by factors such as TWB or ABW use, pathogen MICs, patient CKD history, and specific RRT parameters.

## Electronic supplementary material

Below is the link to the electronic supplementary material.

Supplementary Material 1

## Data Availability

The anonymized data supporting the findings of this study are available upon reasonable request to the corresponding author.

## Abbreviations

ABW: Adjusted body weight
AKI: Acute kidney injury
AUC: Area under the curve
BIC: Corrected bayesian information criterion
CI: Confidence interval
CKD: Chronic kidney disease
Cl: Total clearance
Cld: Dialytic clearance
C_max_: Peak concentration
C_min_: Trough concentration
ICU: Intensive care unit
MIC: Minimum Inhibitory Concentration
RRT: Renal replacement therapy
TBW: Total body weight
