# Supplementary material for: Amikacin use in critically ill patients requiring renal replacement therapy: the AMIDIAL-ICU study
Source: Ann Intensive Care. 2025 Mar 26;15:42. doi: 10.1186/s13613-025-01461-z (PMC11937451; doi:10.1186/s13613-025-01461-z)
Supplement: Supplementary file 1 — Supplementary Material 1 [file 13613_2025_1461_MOESM1_ESM.docx]

**Amikacin use in critically ill patients requiring renal replacement therapy: the AMIDIAL-ICU study**

**Supplemental material**

**Supplemental Table 1.** Characteristics of infection

**Supplemental Table 2.** Characteristics of renal replacement therapy

**Supplemental Table 3.** Population pharmacokinetic parameters of model 1

**Supplemental Table 4.** Population pharmacokinetic parameters of model 2

**Supplemental Table 5.** Fractional target attainment with several amikacin dosing regimens based on total body weight

**Supplemental Table 6.** Fractional target attainment with several amikacin dosing regimens based on adjusted body weight

**Supplemental Figure 1.** Geographic distributions of including centers

**Supplemental Figure 2.** Flow chart

**Supplemental Figure 3.** Goodness-of-fit plots: observations versus predictions for amikacin concentrations [mg/L].

**Supplemental Figure 4.** Diagnostic plots. The figure shows the PWRES, IWRES and NPDE as a function of time and PWRES, IWRES and NPDE as a function of individual and population prediction of amikacin concentration.

**Supplemental Figure 5.** Visual predictive checks (VPC, n=10000 simulations) for amikacin concentrations of the final model.

**Supplemental Figure 6. Probabilities of amikacin pharmacokinetic targets achievement in critically ill patients undergoing renal replacement therapy using adjusted body weight**

**Supplemental Table 1. Characteristics of infection**

| Variables | All patients  (n=111) |
| --- | --- |
| Source of infection, n(%) |  |
| Intraabdominal | 9(8.1) |
| Respiratory | 8(7.2) |
| Urine | 6(5.4) |
| Catheter | 6(5.4) |
| Skin and soft tissue | 2(1.8) |
| Not identified | 80(72.1) |
| Bacteriemia, n(%) | 39(35.1) |
| Identified pathogen, n(%) |  |
| None | 36(32.4) |
| *Escherichia coli* | 13(11.7) |
| *Klebsiella pneumoniae* | 10(9.0) |
| *Pseudomonas aeruginosa* | 10(9.0) |
| *Enterobacter spp.* | 5(4.5) |
| Others | 37(33.3) |
| Antimicrobial combination therapy, n(%) |  |
| Penicillin | 46(41.4) |
| Carbapenem | 45(40.5) |
| Cephalosporin | 39(35.1) |
| Glycopeptide | 14(12.6) |
| Oxazolidinone | 11(9.9) |
| Nitroimidazole | 11(9.9) |
| Fluoroquinolone | 9(8.1) |
| Lipopeptide | 9(8.1) |
| Antifungal therapy | 7(6.3) |
| Macrolide | 3(2.7) |
| Lincosamide | 3(2.7) |
| Others | 5(4.5) |

Variables are presented as number(percentage).

**Supplemental Table 2. Characteristics of renal replacement therapy**

| Variables | All patients  (n=111) |
| --- | --- |
| RRT indication, n(%) |  |
| AKI | 102(91.9) |
| Metabolic acidosis | 51(46.0) |
| Hyperkalemia | 39(35.1) |
| Chronic hemodialysis | 10(9.0) |
| Pulmonary oedema | 8(7.2) |
| Toxic | 8(7.2) |
| Tumor lysis syndrome | 3(2.7) |
| RRT modality, n(%) |  |
| Continuous | 77(69.4) |
| CVVH | 42(60) |
| CVVHDF | 18(25.7) |
| CVVHD | 10(14.3) |
| Blood flow, mL/min | 150(150-200) |
| Predilution effluent, mL/h | 600(0-750) |
| Postdilution effluent, mL/h | 1400(750-1600) |
| Dialysate flow, mL/h | 1500(0-2000) |
| Duration, h | 49(24-67) |
| Intermittent | 34(32.6) |
| Blood flow, mL/min | 225(200-250) |
| Dialysate flow, mL/min | 450(240-500) |
| Duration, h | 4.5(4-7) |

Variables are presented as median(interquartile range) or number(percentage) as appropriate. AKI, acute kidney injury; RRT, renal replacement therapy; CVVH, continuous venovenous hemofiltration; CVVHDF, continuous venovenous hemodiafiltration; CVVHD, continuous venovenous hemodialysis.

**Supplemental Table 3. Population pharmacokinetic parameters of model 1**

| Parameter | Value | Stoch. Approx. | | Bootstrapping (n=1,000) | |
| --- | --- | --- | --- | --- | --- |
|  |  | S.E. | R.S.E.(%) | Median Value^a^ | CI95%^a^ Value |
| *Fixed Effects* | | | | | |
| Cl_pop | 0.68 | 0.16 | 23.08 | 0.68 | [0.29 ; 0.95] |
| Cld_pop | 1.68 | 0.32 | 18.97 | 1.68 | [1.22 ; 2.59] |
| beta_Cld_CKD=Yes | -0.68 | 0.28 | 40.46 | -0.68 | [-1.07 ; -0.11] |
| beta_Cld_Continous RRT modality=Yes | -0.89 | 0.33 | 36.83 | -0.90 | [-2.47 ; -0.26] |
| V1_pop | 22.6 | 1.91 | 8.44 | 22.60 | [18.28 ; 25.24] |
| beta_V1_logtweight | 0.71 | 0.23 | 32.2 | 0.71 | [0.29 ; 1.10] |
| Q_pop | 1.46 | 0.68 | 46.9 | 1.46 | [0.81 ; 3.96] |
| V2_pop | 22.98 | 5.75 | 25.03 | 22.98 | [16.47 ; 44.03] |
| *Standard Deviation of the Random Effects* | | | | | |
| omega_Cl | 0.89 | 0.14 | 15.77 | 0.89 | [0.62 ; 1.29] |
| omega_Cld | 0.37 | 0.13 | 34.07 | 0.37 | [0.18 ; 0.58] |
| omega_V1 | 0.32 | 0.055 | 17.12 | 0.32 | [0.23 ; 0.41] |
| omega_Q | 0.76 | 0.28 | 36.39 | 0.76 | [0.29 ; 1.12] |
| omega_V2 | 0.79 | 0.17 | 21.7 | 0.79 | [0.29 ; 1.37] |
| *Error Model Parameters* | | | | | |
| a | 0.25 | 0.086 | 33.9 | 0.25 | [0.12 ; 0.52] |
| b | 0.16 | 0.026 | 15.8 | 0.16 | [0.05 ; 0.22] |

rse-sa: relative se_sa estimated using recommended stochastic approximation. Population parameter was modelled as follow: log(Cl) = log(Cl_pop) + eta_Cl

log(Cld) = log(Cld_pop) + beta_Cld_CKD*[CKD = Yes] + beta_Cld_ Continous RRT modality*[ Continous RRT modality=Yes] + eta_Cld

log(V1) = log(V1_pop) + beta_V1_logtweight*logtweight + eta_V1

log(Q) = log(Q_pop) + eta_Q

log(V2) = log(V2_pop) + eta_V2. For each parameter. eta is the random effect.

a: Population estimates and their confident interval 95% were calculated using the nonparametric bootstrap (n=1,000).

**Supplemental Table 4. Population pharmacokinetic parameters of model 2**

| Parameter | Value | Stoch. Approx. | | Bootstrapping  (n=1,000) | |
| --- | --- | --- | --- | --- | --- |
|  |  | S.E. | R.S.E.(%) | Median Value^a^ | CI95%^a^ Value |
| *Fixed Effects* | | | | | |
| Cl_pop | 0.43 | 0.14 | 33.29 | 0.43 | [0.02 ; 0.83] |
| Cld_pop | 2.01 | 0.26 | 12.84 | 2.01 | [1.07 ; 2.54] |
| beta_Cld_CKD=Yes | -0.56 | 0.19 | 34.48 | -0.56 | [-1.11 ; -0.01] |
| beta_Cld_Continous RRT modality=Yes | -0.95 | 0.27 | 28.6 | -0.96 | [-2.20 ; -0.04] |
| V1_pop | 22.13 | 1.2 | 5.42 | 22.13 | [19.89 ; 26.41] |
| beta_V1_logtABW | 0.95 | 0.27 | 28.68 | 0.95 | [0.50 ; 1.40] |
| Q_pop | 1.72 | 0.38 | 22.33 | 1.72 | [0.70 ; 2.75] |
| V2_pop | 26.77 | 6.5 | 24.27 | 26.77 | [19.05 ;331.89] |
| *Standard Deviation of the Random Effects* | | | | | |
| omega_Cl | 1.14 | 0.22 | 19.55 | 1.14 | [0.62 ; 1.87] |
| omega_Cld | 0.38 | 0.11 | 28.72 | 0.38 | [0.17 ; 0.61] |
| omega_V1 | 0.33 | 0.047 | 14.04 | 0.33 | [0.19 ; 0.38] |
| omega_Q | 0.56 | 0.16 | 28.52 | 0.55 | [0.28 ; 1.02] |
| omega_V2 | 0.77 | 0.24 | 30.7 | 0.77 | [0.38 ; 1.83] |
| *Error Model Parameters* | | | | | |
| a | 0.28 | 0.091 | 32.82 | 0.28 | [0.02 ; 0.45] |
| b | 0.16 | 0.023 | 14.44 | 0.16 | [0.06 ; 0.25] |

rse-sa: relative se_sa estimated using recommended stochastic approximation. Population parameter was modelled as follow: log(Cl) = log(Cl_pop) + eta_Cl

log(Cld) = log(Cld_pop) + beta_Cld_CKD*[CKD = Yes] + beta_Cld_ Continous RRT modality*[ Continous RRT modality=Yes] + eta_Cld

log(V1) = log(V1_pop) + beta_V1_logtABW*logtABW + eta_V1

log(Q) = log(Q_pop) + eta_Q

log(V2) = log(V2_pop) + eta_V2. For each parameter. eta is the random effect.

a: Population estimates and their confident interval 95% were calculated using the nonparametric bootstrap (n=1,000).

|  | Fractional target attainment (%) for MIC of | | | | | | | | | |
| --- | --- | --- | --- | --- | --- | --- | --- | --- | --- | --- |
|  | 1mg/L | | 2mg/L | | 4mg/L | | 8mg/L | | 16mg/L | |
|  | C_max_/MIC ≥8 | AUC/MIC of ≥75 | C_max_/MIC ≥8 | AUC/MIC of ≥75 | C_max_/MIC ≥8 | AUC/MIC of ≥75 | C_max_/MIC ≥8 | AUC/MIC of ≥75 | C_max_/MIC ≥8 | AUC/MIC of ≥75 |
| *No CKD / Continuous RRT* |  |  |  |  |  |  |  |  |  |  |
| 15mg/kg | 100 | 100 | 99.98 | 98.56 | 94.75 | 71.86 | 27.94 | 7.07 | 0.20 | 0.01 |
| 20mg/kg | 100 | 100 | 100 | 99.65 | 99.29 | 90.04 | 61.94 | 27.60 | 3.13 | 0.22 |
| 25mg/kg | 100 | 100 | 100 | 99.86 | 99.91 | 96.37 | 85.49 | 52.82 | 12.49 | 1.86 |
| 30mg/kg | 100 | 100 | 100 | 99.93 | 99.98 | 98.28 | 94.38 | 72.30 | 27.87 | 7.43 |
| 35mg/kg | 100 | 100 | 100 | 99.99 | 99.98 | 99.44 | 98.24 | 83.82 | 46.06 | 16.33 |
| *No CKD / Intermittent RRT* |  |  |  |  |  |  |  |  |  |  |
| 15mg/kg | 100 | 100 | 99.98 | 99.37 | 94.96 | 90.19 | 29.65 | 28.39 | 0.26 | 0.18 |
| 20mg/kg | 100 | 100 | 100 | 99.83 | 99.32 | 96.86 | 63.38 | 60.36 | 3.55 | 2.99 |
| 25mg/kg | 100 | 100 | 100 | 99.9 | 99.91 | 98.71 | 86.07 | 80.78 | 13.65 | 12.20 |
| 30mg/kg | 100 | 100 | 100 | 99.95 | 99.98 | 99.17 | 94.58 | 89.96 | 29.39 | 28.77 |
| 35mg/kg | 100 | 100 | 100 | 99.99 | 99.99 | 99.75 | 98.3 | 94.26 | 47.78 | 45.27 |
| *CKD /*  *Continuous RRT* |  |  |  |  |  |  |  |  |  |  |
| 15mg/kg | 100 | 100 | 99.98 | 99.32 | 94.96 | 88.12 | 29.35 | 23.27 | 0.26 | 0.10 |
| 20mg/kg | 100 | 100 | 100 | 99.83 | 99.32 | 96.20 | 63.16 | 54.47 | 3.49 | 1.97 |
| 25mg/kg | 100 | 100 | 100 | 99.90 | 99.91 | 98.48 | 94.56 | 77.05 | 13.41 | 9.06 |
| 30mg/kg | 100 | 100 | 100 | 99.95 | 99.98 | 99.13 | 94.56 | 87.82 | 29.09 | 23.50 |
| 35mg/kg | 100 | 100 | 100 | 99.99 | 99.99 | 99.73 | 98.29 | 93.23 | 47.61 | 39.27 |
| *CKD /*  *Intermittent RRT* |  |  |  |  |  |  |  |  |  |  |
| 15mg/kg | 100 | 100 | 99.98 | 99.50 | 95.95 | 93.93 | 30.16 | 41.81 | 0.27 | 0.78 |
| 20mg/kg | 100 | 100 | 100 | 99.87 | 99.33 | 97.60 | 63.80 | 71.21 | 3.72 | 7.36 |
| 25mg/kg | 100 | 100 | 100 | 99.91 | 99.91 | 98.92 | 86.31 | 86.27 | 13.98 | 22.46 |
| 30mg/kg | 100 | 100 | 100 | 99.95 | 99.98 | 99.36 | 94.66 | 92.99 | 29.95 | 42.32 |
| 35mg/kg | 100 | 100 | 100 | 100 | 99.99 | 99.80 | 98.34 | 95.95 | 48.35 | 58.65 |

**Supplemental Table 5. Fractional target attainment with several amikacin dosing regimens based on total body weight**

Monte Carlo simulations and probability of target achievement for various amikacin doses for patients with or without chronic kidney disease (CKD) history undergoing continuous or intermittent renal replacement therapy (RRT) for various minimum inhibitory concentrations (MIC). Acceptable situations, defined as probabilities of target achievements ≥90%, are highlighted in green. Unacceptable situations are highlighted in red. C_max_, maximum concentration; AUC, area under the curve.

**Supplemental Table 6. Fractional target attainment with several amikacin dosing regimens based on adjusted body weight**

|  | Fractional target attainment (%) for MIC of | | | | | | | | | |
| --- | --- | --- | --- | --- | --- | --- | --- | --- | --- | --- |
|  | 1mg/L | | 2mg/L | | 4mg/L | | 8mg/L | | 16mg/L | |
|  | C_max_/MIC ≥8 | AUC/MIC of ≥75 | C_max_/MIC ≥8 | AUC/MIC of ≥75 | C_max_/MIC ≥8 | AUC/MIC of ≥75 | C_max_/MIC ≥8 | AUC/MIC of ≥75 | C_max_/MIC ≥8 | AUC/MIC of ≥75 |
| *No CKD / Continuous RRT* |  |  |  |  |  |  |  |  |  |  |
| 15mg/kg | 100 | 99.79 | 99.92 | 94.99 | 82.98 | 41.82 | 11.30 | 0.55 | 0.01 | 0.00 |
| 20mg/kg | 100 | 99.93 | 100 | 98.67 | 96.94 | 73.68 | 37.56 | 6.31 | 0.49 | 0.00 |
| 25mg/kg | 100 | 99.95 | 100 | 99.36 | 99.46 | 88.72 | 66.55 | 21.51 | 3.50 | 0.03 |
| 30mg/kg | 100 | 99.95 | 100 | 99.65 | 99.87 | 94.78 | 83.44 | 42.82 | 11.21 | 0.57 |
| 35mg/kg | 100 | 99.99 | 100 | 99.85 | 99.97 | 97.37 | 92.98 | 60.07 | 23.27 | 2.38 |
| *No CKD / Intermittent RRT* |  |  |  |  |  |  |  |  |  |  |
| 15mg/kg | 100 | 99.79 | 99.82 | 98.26 | 83.97 | 79.86 | 12.49 | 8.56 | 0.04 | 0.00 |
| 20mg/kg | 100 | 99.97 | 100 | 99.41 | 97.04 | 93.07 | 39.27 | 34.70 | 0.73 | 0.17 |
| 25mg/kg | 100 | 99.96 | 100 | 99.60 | 99.49 | 96.85 | 68.23 | 63.16 | 4.25 | 1.97 |
| 30mg/kg | 100 | 99.95 | 100 | 99.79 | 99.89 | 98.14 | 84.43 | 80.23 | 12.75 | 8.86 |
| 35mg/kg | 100 | 100 | 100 | 99.86 | 99.97 | 99.04 | 93.36 | 88.40 | 25.29 | 20.20 |
| *CKD /*  *Continuous RRT* |  |  |  |  |  |  |  |  |  |  |
| 15mg/kg | 99.92 | 99.75 | 99.98 | 97.74 | 83.76 | 69.89 | 12.16 | 3.70 | 0.04 | 0.00 |
| 20mg/kg | 100 | 99.96 | 100 | 99.27 | 97.01 | 89.53 | 38.81 | 22.06 | 0.68 | 0.04 |
| 25mg/kg | 100 | 99.96 | 100 | 99.55 | 99.47 | 95.63 | 67.73 | 49.07 | 3.97 | 0.69 |
| 30mg/kg | 100 | 99.95 | 100 | 99.76 | 99.88 | 97.68 | 84.15 | 70.40 | 12.26 | 3.75 |
| 35mg/kg | 100 | 100 | 100 | 99.86 | 99.97 | 98.88 | 93.26 | 82.37 | 24.77 | 10.91 |
| *CKD /*  *Intermittent RRT* |  |  |  |  |  |  |  |  |  |  |
| 15mg/kg | 100 | 99.79 | 99.92 | 98.53 | 84.25 | 86.24 | 12.85 | 17.64 | 0.05 | 0.02 |
| 20mg/kg | 100 | 99.97 | 100 | 99.54 | 97.07 | 95.95 | 39.83 | 49.48 | 0.77 | 0.80 |
| 25mg/kg | 100 | 99.96 | 100 | 99.68 | 99.50 | 97.48 | 68.61 | 74.83 | 4.44 | 5.25 |
| 30mg/kg | 100 | 99.95 | 100 | 99.81 | 99.89 | 98.44 | 84.62 | 86.59 | 13.15 | 17.25 |
| 35mg/kg | 100 | 100 | 100 | 99.98 | 99.97 | 99.18 | 93.52 | 91.82 | 25.86 | 32.91 |

Monte Carlo simulations and probability of target achievement for various amikacin doses for patients with or without chronic kidney disease (CKD) history undergoing continuous or intermittent renal replacement therapy (RRT) for various minimum inhibitory concentrations (MIC). Acceptable situations, defined as probabilities of target achievements ≥90%, are highlighted in green. Unacceptable situations are highlighted in red. C_max_, maximum concentration; AUC, area under the curve.


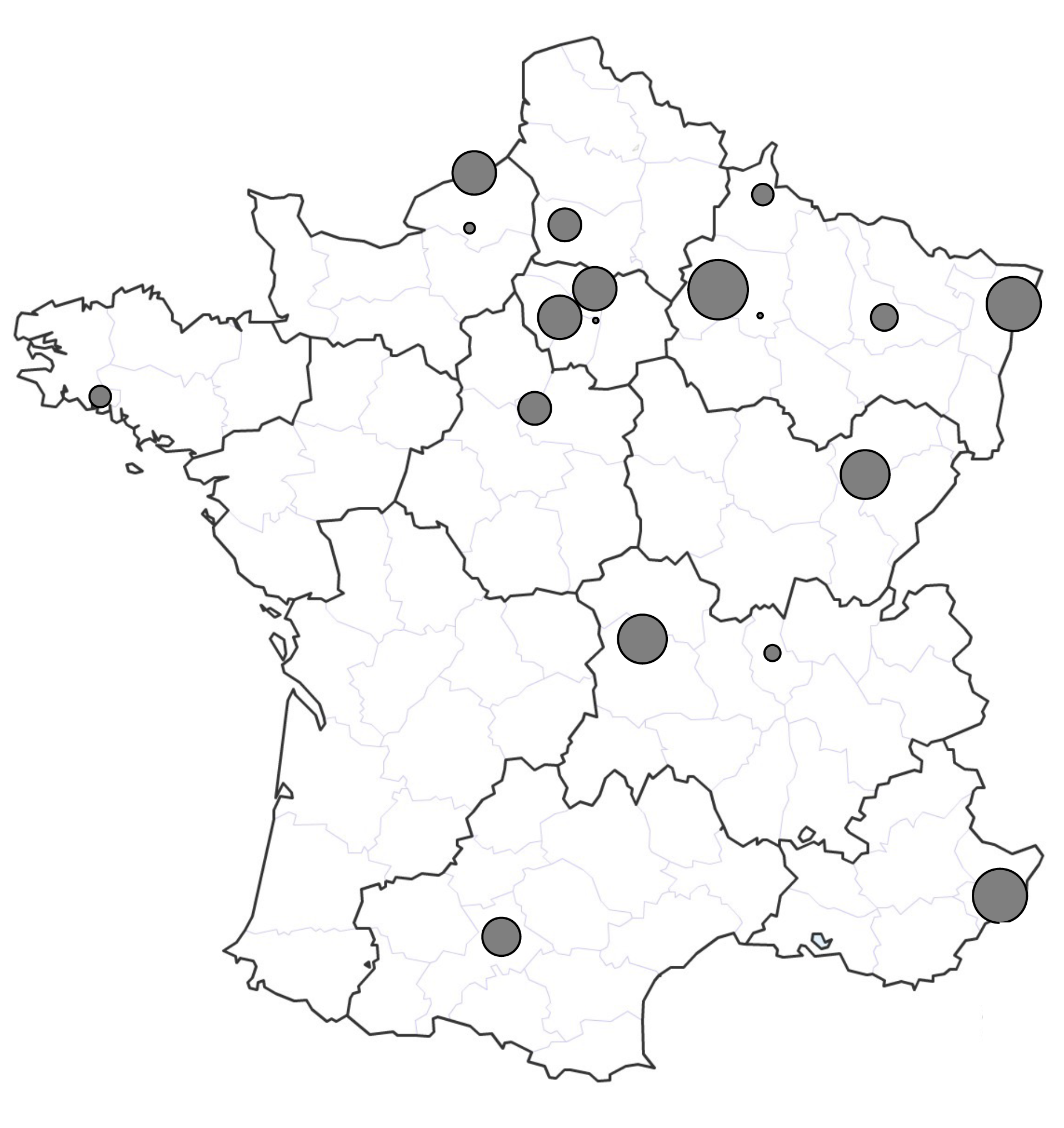


**Supplemental Figure 1. Geographic distributions of including centers**

Grey dots represent centers participating in the study. Size of the dot indicates the number of included patients per center (maximum 10 consecutive patients).

**Supplemental Figure 2. Flow chart**

ICU, intensive care unit; RRT, renal replacement therapy.


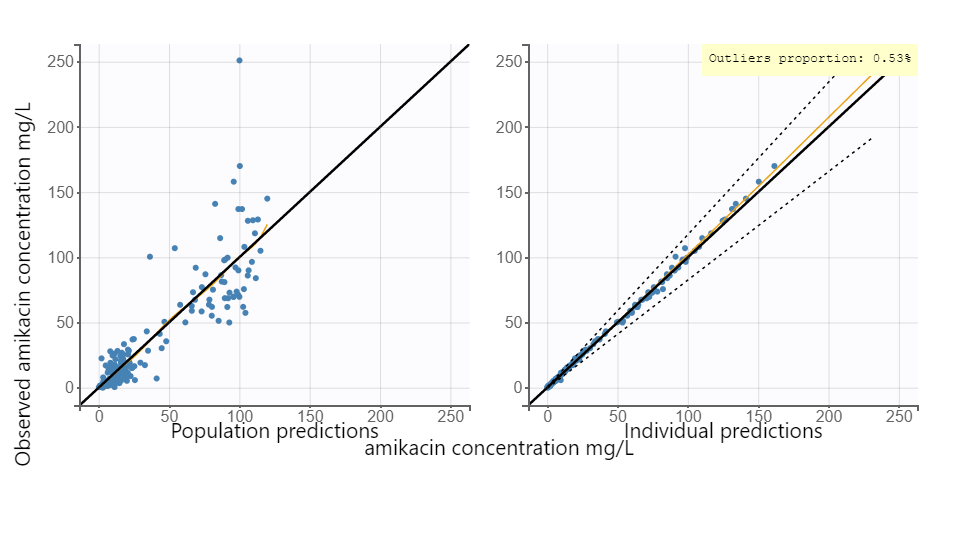


**Supplemental Figure 3.** Goodness-of-fit plots: observations versus predictions for amikacin concentrations [mg/L]. The black line shows the identity line.


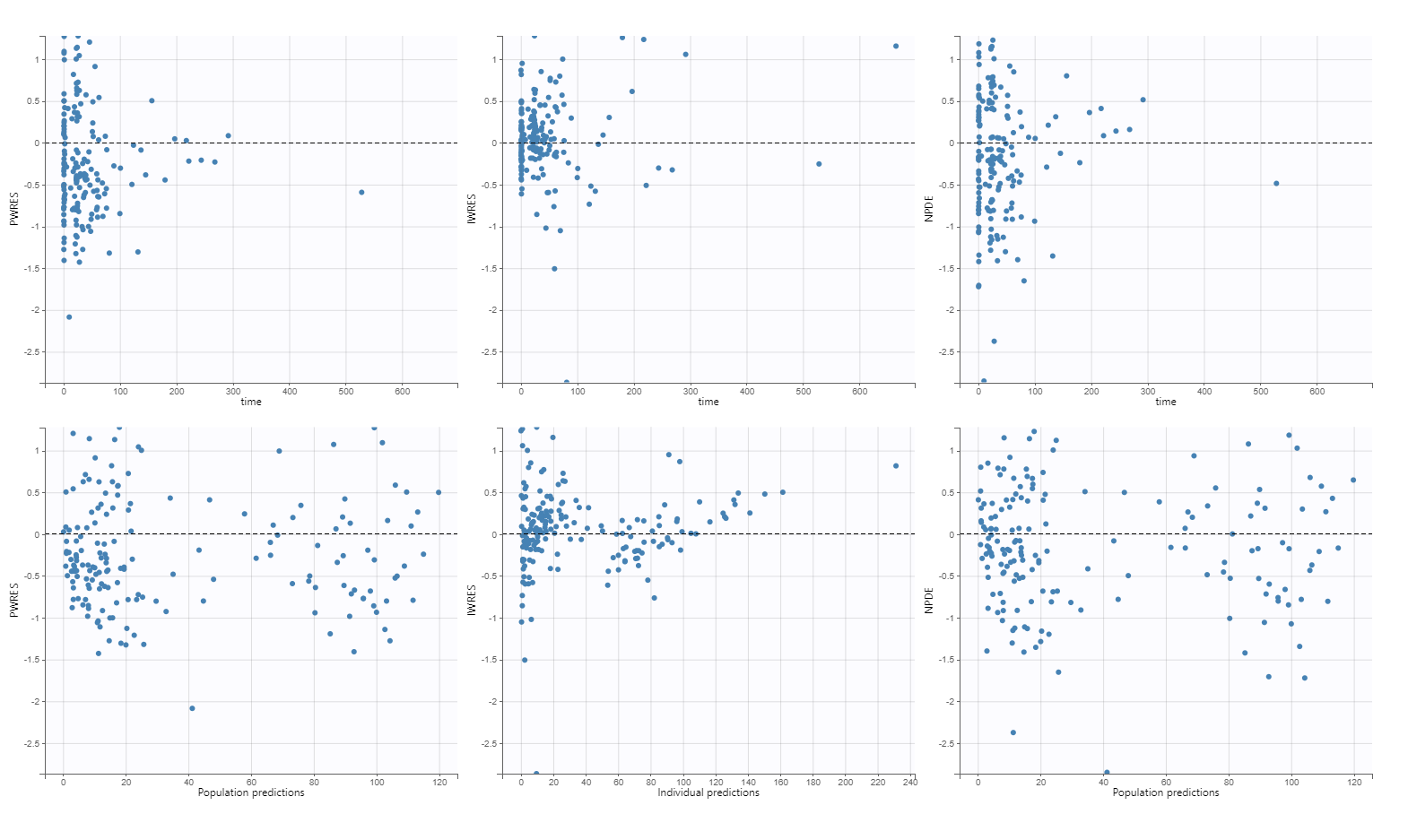


**Supplemental Figure 4.** Diagnostic plots. The figure shows the PWRES, IWRES and NPDE as a function of time and PWRES, IWRES and NPDE as a function of individual and population prediction of amikacin concentration.


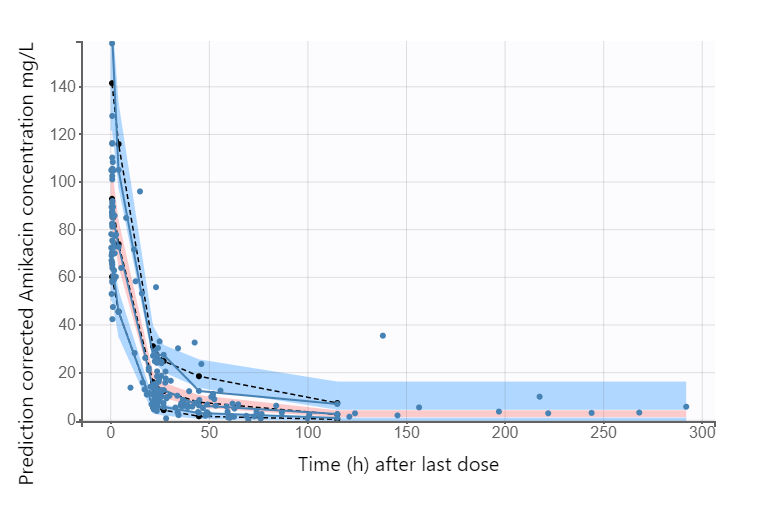


**Supplemental Figure 5.** Visual predictive checks (VPC, n=10000 simulations) for amikacin concentrations of the final model. Blue dots represent observations. Blue lines represent the 10th, 50th, and 90th percentiles of observed concentrations, blue areas the prediction intervals of the 10th and 90th percentiles, light red areas the prediction interval of the median.


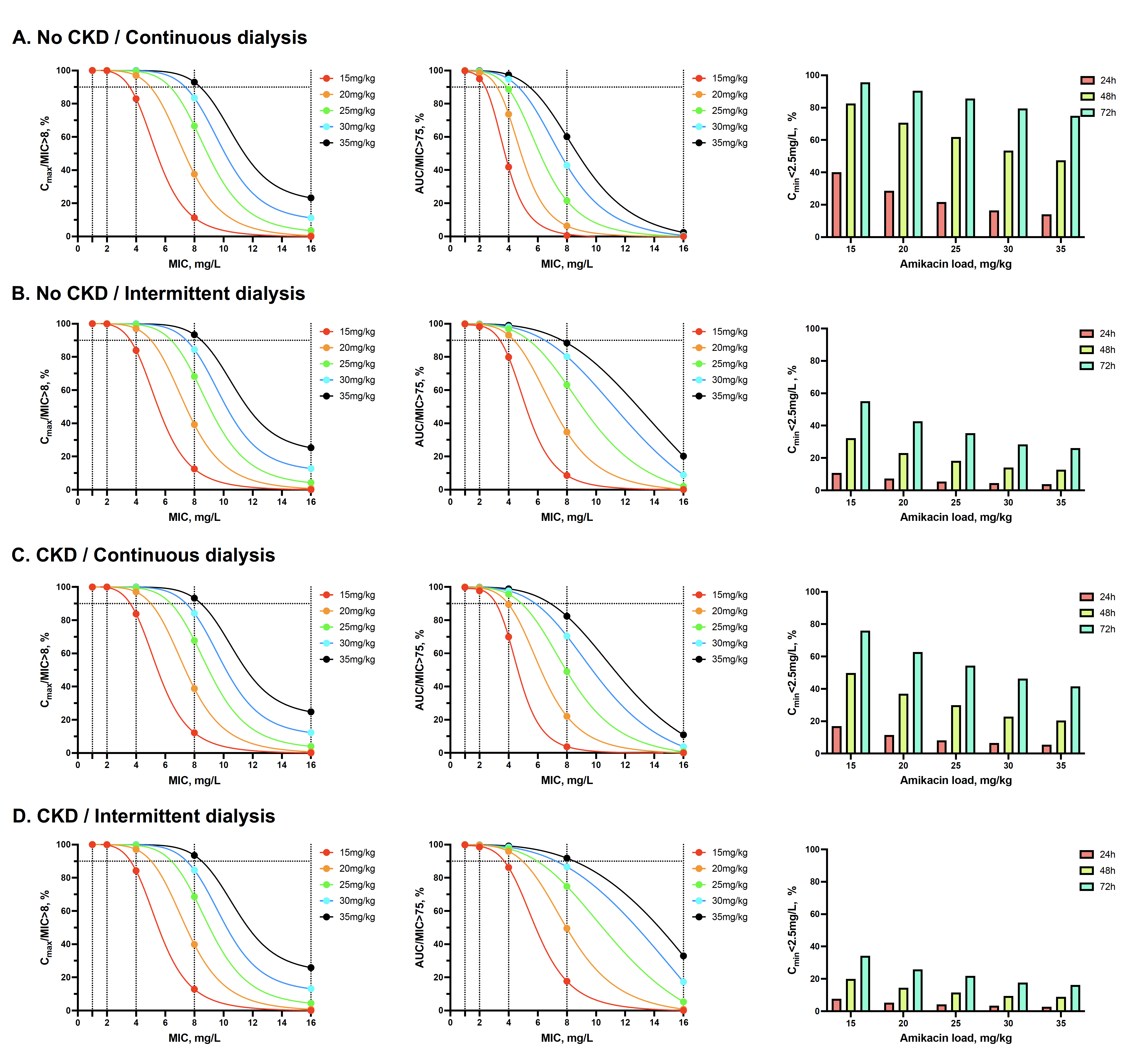


**Supplemental Figure 6. Probabilities of amikacin pharmacokinetic targets achievement in critically ill patients undergoing renal replacement therapy using adjusted body weight**

The probabilities to achieve a maximum concentration (C_max_) / minimum inhibitory concentration (MIC) ratio> 8, an area under the curve (AUC) within the first 24 hours / MIC> 75 and a minimum concentration< 2.5mg/L at 24-48-72h in patients with a history of chronic kidney disease (CKD) treated with continuous (**A**) or intermittent (**B**) dialysis and patients without history of CKD treated with continuous (**C**) or intermittent (**D**) dialysis are depicted, based on Monte Carlo simulation.
